# Coordinating antigen cytosolic delivery and danger signaling to program potent cross-priming by micelle-based nanovaccine

**DOI:** 10.1038/celldisc.2017.7

**Published:** 2017-04-04

**Authors:** Zhida Liu, Chang Zhou, Yan Qin, Zihao Wang, Luyao Wang, Xiuli Wei, Yinjian Zhou, Qicheng Li, Hang Zhou, Wenjun Wang, Yang-Xin Fu, Mingzhao Zhu, Wei Liang

**Affiliations:** 1 Key Laboratory of Infection and Immunity, Institute of Biophysics, Chinese Academy of Sciences, Beijing, China; 2 University of Chinese Academy of Sciences, Beijing, China; 3 The Department of Pathology and Immunology, UT Southwestern Medical Center, Dallas, TX, USA; 4 Protein and Peptide Pharmaceutical Laboratory, Institute of Biophysics, Chinese Academy of Sciences, Beijing, China

**Keywords:** micelle, cancer therapeutic vaccine, antigen cytosolic delivery, cross priming, lymph node targeting, dendritic cell, CTL

## Abstract

Although re-activating cytotoxic T-cell (CTLs) response inside tumor tissues by checkpoint blockade has demonstrated great success in tumor immunotherapy, active induction of efficient endogenous CTL response by therapeutic vaccines has been largely hampered by inefficient cytosolic delivery of antigens and coordinated activation of dendritic cells (DCs) in lymph nodes. Here we show that polyethylene glycol-phosphatidylethanolamine (PEG-PE) micelles transform soluble peptides into α-helix to enable their efficient cytosolic delivery. The same PEG-PE micelles also serve as chaperon of TLR4 signaling to coordinate its adjuvant effect on the same DCs. Furthermore, these nanovaccines effectively target lymph node DCs. Thus, PEG-PE micelle vaccines program at multiple key aspects for inducing strong CTL responses and build up a foundation for combinational tumor therapy.

## Introduction

The importance of cytotoxic T-lymphocyte (CTL) response has been increasingly acknowledged in a series of tumor therapeutic approaches, such as chemotherapy, radiation therapy, antibody mediated targeting therapy and so on [1–4]. It has been further exemplified with recent great success of checkpoint blockade [5, 6]. All these therapeutic methods generate CTL response passively or depend on the pre-existing CTL [1, 7]. To actively generate efficient endogenous CTL response by vaccines is an attractive goal in tumor control. However, the efficacies of therapeutic vaccines have been less impressive. Active induction of efficient endogenous CTL response by therapeutic vaccines has been largely hampered by lacking of coordinating cytosolic delivery of antigens and toll-like receptor signaling on the same antigen presenting cells (APCs) in lymph nodes (LNs).

For exogenous soluble protein antigens to induce an efficient CTL response, efficient cytosolic antigen delivery into APCs for cross-presentation is a key step [8]. Enlightened by the studies of viral membrane fusion proteins, a lot of cell penetrating peptides (CPPs) have been identified and shown advantages for cytosolic delivery of protein antigens when they are fused together [9, 10]. However, CPP-fusion may alter the subcellular localization of the fusion protein and impair its function [11]. Alpha-helix has been found to be the key structure mediating membrane fusion and cytosolic delivery of polypeptides [12–16]. But many soluble antigenic peptides are non-alpha-helix [17]. Therefore, for this type of antigens, it is challenging to transform them into α-helix for efficient cytosolic delivery.

In addition to antigen cytosolic delivery into APCs, the same APCs need to be coordinately activated by adjuvants. Monophosphoryl lipid A (MPLA), a detoxified derivative of lipid A from lipopolysaccharide (LPS), has been approved by FDA and widely used for vaccine studies. Although safer than LPS, its adjuvant activity is also significantly reduced [18]. Therefore, further improvement of its adjuvant effect is still required.

Many types of nanomaterials have been intensively investigated for the co-delivery of antigens and adjuvants targeting specialized anatomical location, such as lymph nodes, and specialized cells, such as DCs, and demonstrated improved DC activation, antigen presentation and therapeutic T-cell response [19–26]. However, a nanoparticle system that enables direct cytosolic antigen delivery into LN DCs has not been reported previously. How to improve the activity of MPLA in the nanopariticle vaccine also remains an issue.

PEG-PE consists of both hydrophobic phosphatidylethanolamine (PE) and hydrophilic polyethylene glycol (PEG). Polymeric nanomicelle system based on the amphiphilic PEG-PE molecule was initially developed and characterized for small molecule drugs cytosolic delivery into tumor cells [27–31]. In this study, we designed PEG-PE micelle vaccines incorporating both antigen peptides and MPLA for co-delivery. PEG-PE micelles converted non-α-helical peptides into α-helix and obtained efficient cytosol antigen delivery. In addition, PEG-PE micelles served as a chaperon to MPLA for TLR signaling and DC activation. Therefore, PEG-PE micelle-based vaccine design enabled efficient co-delivery of tumor antigens and MPLA adjuvant into the same APC and induced dramatically increased CTL response. These nanovaccines compound with antigens and adjuvant are shown to have impressive therapeutic antitumor effect in several tumor models and lay out foundation for potent combinational therapy for established tumors.

## Results

### PEG-PE micelles transform non-α-helical peptides into α-helix peptides for cytosolic delivery

**Figure 1:**
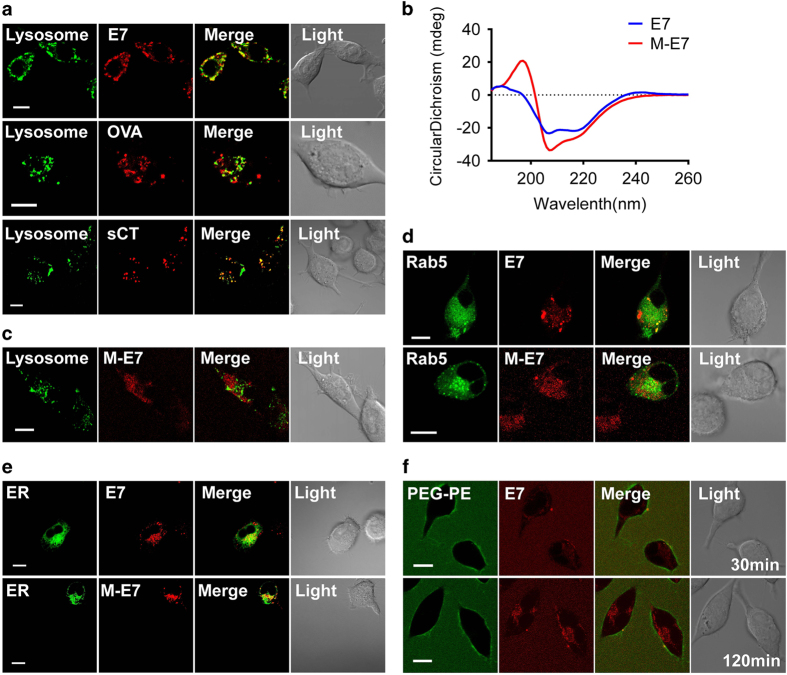
PEG-PE micelle efficiently delivers the antigens into the cell cytosol. (**a**) DC2.4 cells were incubated with 20 μm RhB-labeled E7, OVA or sCT peptides, respectively for 30 min. Subsequently, LysoTrackerGreen was used to track lysosomes, and then the co-localization of intracellular RhB-E7 and lysosomes were analyzed by confocal scanning microscope. (**b**) Secondary structure of E7 peptide and M-E7 was determined by Circular dichroism (CD) spectroscopy. (**c**) 20 μm PEG-PE micellized RhB-E7 (M-E7) was added to DC2.4 cells for 30 min. The co-localization of intracellular RhB-E7 and lysosomes were analyzed. (**d**) DC2.4 cells were transiently transfected with EGFP-Rab5 to label the early endosomes, and then incubated with 20 μm RhB-labeled E7 or M-E7, respectively for 15 min. The co-localization of intracellular RhB-E7 and early endosomes were analyzed. (**e**) DC2.4 cells were incubated with 20 μm RhB-labeled E7 or M-E7, respectively for 120 min. Subsequently, ER Tracker Green was used to track ERs, the co-localization of intracellular RhB-E7 and ERs were analyzed 20 min later. (**f**) DC2.4 cells were incubated with 20 μm PEG-PE micellized E7 peptide (FITC-labeled PEG-PE, RhB-labeled E7 peptide) for a period of 2 h. Cellular internalization and distribution of both PEG-PE (green) and RhB (red) were detected by confocal scanning microscope imaging. Scale bar, 10 μm. Data represent three independent experiments. DC, dendritic cell; PEG-PE, polyethylene glycol-phosphatidylethanolamine; RhB, Rhodamine B; sCT, salmon calcitonin peptide.

Cytosolic delivery of antigen is favorable for CTL priming through cross-presentation pathway. However, many commonly studied antigenic peptides cannot be directly and efficiently delivered into cytosol. Here we tested several peptides *in vitro* for their cytosolic delivery property. Briefly, HPV16 E7_43-62_ peptide, OVA_250-264_ peptide and salmon calcitonin peptide (sCT, a peptide drug) were labeled Rhodamine B (RhB) and incubated with DC2.4 for 2 h. The presence and the location of these peptides were determined by confocal imaging. Significant amount of these peptides were uptaken by DC2.4 cells. In stark contrast, most E7 and sCT peptides were contained within lysosomes (Figure 1a, top and bottom panel), while OVA peptide was largely outside lysosomes (Figure 1a, middle panel), suggesting a cytosolic presence. Secondary structure analysis showed that OVA peptide is α-helix peptide (Supplementary Figure S1A), while HPV16 E7 (Supplementary Figure S1B) and sCT peptides (Supplementary Figure S1C) are both non-α-helical peptides. These data support the general importance of α-helix peptides for cytosolic trafficking.

We have recently demonstrated that PEG-PE micelles could assist non-native protein refolding into α-helix structure and avoid protein aggregation [28]. This prompted us to test whether PEG-PE micelle can transform non-α-helical tumor peptides for cell membrane fusion and cytosolic delivery. To this end, E7 peptide was encapsulated into the PEG-PE micelle. The circular dichroism (CD) spectroscopy result showed that the micelle encapsulating E7 (M-E7) had a more obvious α-helical structure characteristic than the free form (Figure 1b). To investigate whether this transformation would improve its membrane translocation ability, E7 and PEG-PE were labeled with different fluorescent dyes, respectively and incubated with DC2.4. Surprisingly, transformed M-E7 was largely localized in the cytosol without any co-localization with lysosomes or endosomes (Figure 1c and d, Supplementary Figure S1D). In addition, more E7 peptide was colocalized with ER when encapsulated by PEG-PE micelle, which will further promote cytosolic antigens to be cross-presented by MHC-I molecules (Figure 1e). In contrast to the cytosolic delivery of E7 peptide, PEG-PE retained on the cell membrane until 2 h (Figure 1f), suggesting a release of antigen payload from PEG-PE micelles at the cell membrane. The same effect of transformation and cytosolic delivery of PEG-PE was also seen with another non-α-helix peptide, sCT (Supplementary Figure S1E and F). Together, these data suggest that PEG-PE micelle can transform non-α-helix peptides to be more α-helical, and bring them for transmembrane delivery, ready for proper cytosolic antigen process, a pre-conditioning for antigen presentation to CD8^+^ T cells.

### PEG-PE micelles chaperon MPLA for enhanced TLR signaling and APC function

**Figure 2:**
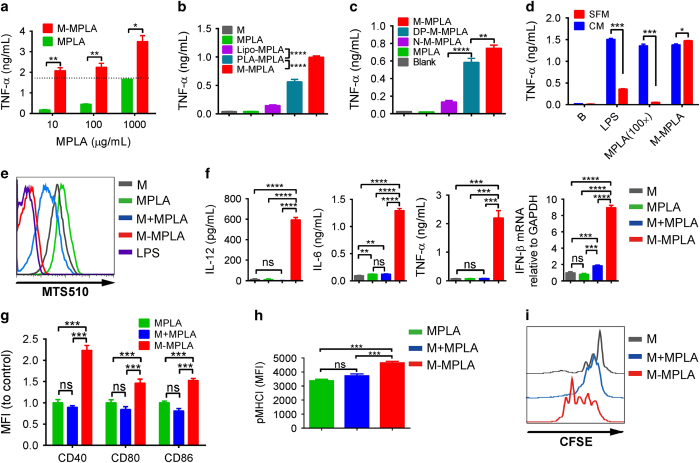
PEG-PE micellized formulation significantly enhances MPLA’s adjuvant efficacy for co-stimulation. Raw264.7 cells were treated with different dose and formulations of MPLA for 2 h. Then the secreted TNF-α was determined by ELISA. (**a**) Cells were treated with different dose of free MPLA or M-MPLA. (**b**) Cells were treated with 100 ng ml^−1^ of different MPLA as indicated, including MPLA liposome (Lipo-MPLA), PEG-PLA micellized MPLA (PLA-MPLA), PEG-PE (M) or (**c**) PEG_2000_-DPG micellized MPLA (DP-M-MPLA), PEG_2000_-L-DPG micellized MPLA (N-M-MPLA). (**d**) Cells were treated with normal saline (B), LPS (100 ng ml^−1^), MPLA (10 μg ml^−1^) or M-MPLA (100 ng ml^−1^ MPLA) in serum-free medium (SFM) or complete medium (CM), respectively. (**e**) The TLR4–MD2 heterotetramer formation was detected on Raw264.7 cells with different MPLA formulations or LPS treatment. (**f**) BMDCs were treated with 100 ng ml^−1^ of different MPLA formulations for 24 h (protein level) or 1 h (RNA level), then the cytokines were determined by ELISA or real-time PCR. Data above are shown as mean±s.d. (*n*=4). (**g**) Splenocytes were incubated with different formulations of MPLA (100 ng ml^−1^) for 12 h. Then the CD11c^+^ MHCII^+^ DCs were gated for CD40, CD80 and CD86 detection. (**h**) Splenocytes were incubated with 100 ng ml^−1^ different MPLA in the presence of 7 μm Pal-OVA for 16 h. The pMHCI on cell surface was detected. The MFI of above data was shown as mean±s.d. (*n*=8). (**i**) The sorted DCs pretreated with 5 μg ml^−1^ Pal-OVA in the presence of M+MPLA or M-MPLA (20 ng ml^−1^) for 16 h, then the CFSE-labeled OT1 T cells were added. Four days later, the CFSE dilution on OT1 T cells was analyzed. *P<0.05; **P<0.01; ***P<0.001; ****P<0.0001. BMDC, bone marrow–derived DC; DC, dendritic cell; LPS, lipopolysaccharide; MFI, mean fluorescent intensity; MPLA, monophosphoryl lipid A; NS, not significant; PEG-PE, polyethylene glycol-phosphatidylethanolamine; RhB, Rhodamine B.

Using proper adjuvant to activate the same antigen carrying DC is critical for antigen process and presentation to induce potent CTLs. According to the encapsulating features of PEG-PE micelles, MPLA with its hydrophobic property was selected. MPLA is a detoxified form of LPS and has been proved by FDA for vaccines. To address whether PEG-PE micellization can influence MPLA adjuvant activity, RAW264.7 cells were stimulated with MPLA or PEG-PE micellized MPLA. Surprisingly, PEG-PE micellization was found to dramatically enhance the MPLA activity as determined by TNF-α production (Figure 2a). A total 10 ng ml^−1^ of MPLA loaded in the PEG-PE micelle generated a comparable level of TNF-α as 1 000 ng ml^−1^ of MPLA did in the free form. When compared with other nanoparticle formations, such as liposome and PEG_2000_-PLA micelle, PEG-PE micelle also demonstrated significant advantages (Figure 2b). Thus, in addition to cytosolic antigen delivery, PEG-PE micelle may have unique physiochemical features conferring its MPLA activity enhancing effect on the same APCs.

To study the mechanisms, we first compared the chemical structure of PEG-PE with that of PEG_2000_-PLA, a formation with similar structure but less enhancing effect. We noticed that PEG-PE molecule has a negatively charged phosphorus group in the middle of the tow block but PEG_2000_-PLA has not. The negatively charged phosphorus may ensure MPLA uniform dispersion in the PEG-PE micelle. To test this hypothesis, we synthesized two PEG-PE analogs: PEG_2000_-DPG without negative charge (Supplementary Figure S2A and B) and PEG_2000_-L-DPG with positively charged nitrogen (Supplementary Figure S2C and D). Interestingly, MPLA encapsulated in the PEG_2000_-DPG micelle (DP-M-MPLA) showed attenuated TNF-α simulating ability compared with MPLA in PEG-PE micelles (Figure 2c). MPLA in PEG_2000_-L-DPG micelle (N-M-MPLA) showed further reduction of the TNF-α stimulating ability (Figure 2c). This is likely due to the difficult release of MPLA for TLR4 binding because of the strong charge–charge interaction between PEG_2000_-L-DPG and MPLA in the micelles. These data further support the importance of the phosphorus group of PEG-PE for MPLA activity enhancing effect.

Both MPLA and LPS have very low critical micelle concentration to tend to form the micelle at 10^−9^ mol concentration [32, 33]. Only their monomers can bind to the TLR4/MD-2 heterodimers, and after binding, two TLR4/MD-2 heterodimers form the TLR4/MD-2 heterotetramers for signaling transduction [34, 35]. As a chaperon, LBP in serum can induce disassembly of MPLA and LPS, and then transfer them with CD14 to their receptors (Supplementary Figure S3A; [36]). In the serum-free medium (without LBP) or addition of LBP-blocking peptide, MPLA and LPS could not efficiently stimulate macrophage to secret TNF-α. Unexpectedly, the adjuvant activity of MPLA encapsulated in PEG-PE micelle was not affected by serum-free medium or LBP blocking (Figure 2d and Supplementary Figure S3B), suggesting an LBP-independent TLR4 activation. In consistence, PEG-PE encapsulated MPLA, similar to LPS, but not other formations, dramatically downregulated MTS510 mAb reactive surface TLR4/MD-2 complex (Figure 2e), indicating an efficient MPLA-TLR4–MD-2 complex formation [37–40]. Thus, PEG-PE micelles facilitate more efficient MPLA-TLR4–MD-2 complex formation likely by dispersing of MPLA aggregation in an LBP-independent manner. PEG-PE micelles serve as chaperon for MPLA.

To further determine whether PEG-PE micelle-encapsulated MPLA (M-MPLA) can better promote APC functions, bone marrow–derived DCs were used for measurement of their co-stimulation and antigen presentation. The proinflammatory cytokines (TNF-α, IL-6, IL-12p70 and IFN-β) expression induced by M-MPLA were dramatically increased comparing with free MPLA and simply physically mixed MPLA with PEG-PE micelle (M+MPLA; Figure 2f). Flow cytometry analysis demonstrated significantly higher expression of maturation markers on the M-MPLA-treated DCs (Figure 2g and Supplementary Figure S3C). In addition, direct measurement of the complex of MHC-I and peptide (SIINFEKL; pMHCI) on DC surface indicated enhanced antigen cross-presentation by M-MPLA (Figure 2h and Supplementary Figure S3D). T-cell proliferation assay further demonstrated the superior capability of M-MPLA stimulated DCs for T-cell activation and proliferation (Figure 2i). Therefore, M-MPLA is a potent adjuvant to activate DCs for T-cell priming.

### Construction of PEG-PE micelle-based dual-deliverable and LNs targeting therapeutic vaccines

**Figure 3:**
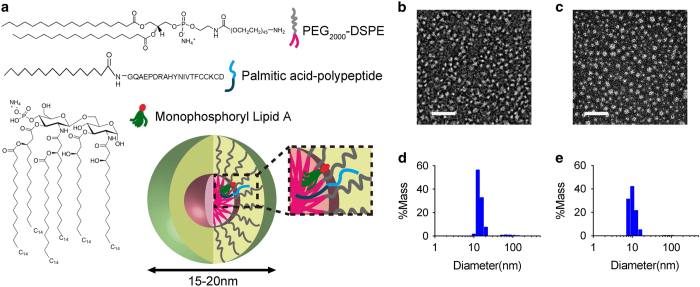
Design of cancer vaccine based on PEG-PE micelle. (**a**) Schematic diagram of self-assembly micelle consist of PEG-PE, palmitoylated polypeptide and MPLA. Upon encapsulation in micelles, the hydrophobic palmitic acid of palmitoylated polypeptide and MPLA can be inserted into the hydrophobic core of the micelles. Transmission electron microscopy (TEM) image of empty PEG-PE micelles (**b**) and micelle vaccine encapsulating the polypeptide antigen and MPLA (**c**). Scale bar, 50 nm. Representative size distribution of micelle vaccine (**d**) and empty PEG-PE micelles (**e**) were measured by dynamic light scattering (DLS) analysis. Data represent two or three independent experiments. MPLA, monophosphoryl lipid A; PEG-PE, polyethylene glycol-phosphatidylethanolamine.

The previous data demonstrated that PEG-PE micelle may be a unique vehicle suitable for both antigen and MPLA adjuvant delivery for vaccine purpose. To induce a strong CD8^+^ T-cell immune response, both antigen and adjuvant are ideally synchronously captured by the same APCs [41–43]. Therefore, we developed an approach to concurrently encapsulate antigen peptides and MPLA adjuvant. To increase the stability of peptides in the micelle, a palmitic acid was used to conjugate to the N-terminal of peptide to enhance the hydrophobic interaction between the core of the micelle and peptide lipid acyl tail. The hydrophobic property of MPLA enables itself to be entrapped into the PEG-PE micelle easily. Film-rehydration method was employed to facilitate the PEG-PE micellar vaccine auto-assembling and encapsulating the antigen peptide and MPLA (Figure 3a). The morphology of micelle observed by transmission electron microscopy showed that incorporation of polypeptide and MPLA did not perturb the geometry of PEG-PE micelles (Figure 3b: empty micelles, Figure 3c: vaccine micelles). Dynamic light scattering revealed that vaccine micelles had a slightly increased diameter (18±5 nm; Figure 3d) compared with empty micelles (15±5 nm; Figure 3e). Thus, PEG-PE micelle-based vaccine auto-assemble into uniform spherical nanoparticles in an optimal size for LN-targeted delivery [44].

**Figure 4:**
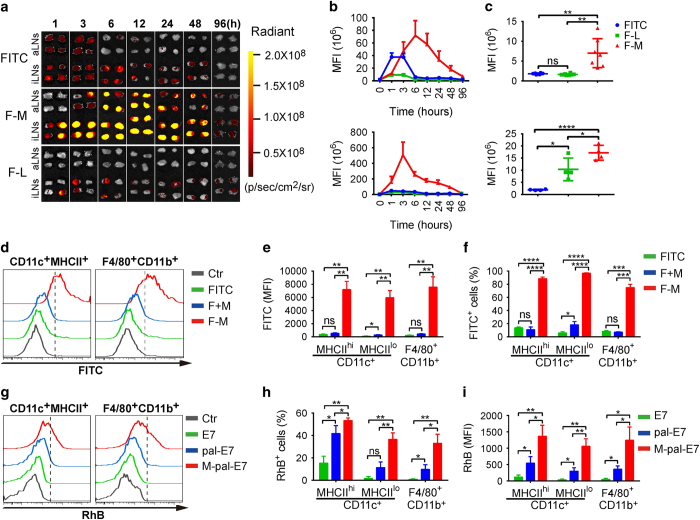
Extensive delivery of micelle to both LN resident and migratory DCs. (**a**–**f**) C57BL/6 mice were subcutaneously injected with different FITC formulations (FITC, FITC-labeled PEG-PE micelle, FITC-labeled liposome) at tail base, the drainage of FITC from injection sites into DLNs and FITC captured by APCs were detected, respectively. (**a**) The accumulation of different FITC formulations in DLNs were monitored at indicated time points (from 0 to 96 h post injection, the axillary LNs are referred as ‘aLNs’ and the inguinal LNs are referred as ‘iLNs’). The fluorescent intensity of axillary LNs (**b**, top panel) and inguinal LNs (**b**, bottom panel) was measured and quantified using the Living Image 4.4 software. The MFI of 96 h post injection is shown (**c**). Data are shown as mean±s.d. (*n*=4–8 LNs per group). (**d**–**f**) The FITC^+^ DCs and macrophages in DLNs were determined by flow cytometry at 24 h post injection. The frequencies (**e**) and MFI (**f**) were analyzed, respectively. The statistical results are shown as mean±s.d. (*n*=4). (**g**) C57BL/6 mice were subcutaneously injected with different formulations of RhB-labeled E7 polypeptide (E7, pal-E7 and M-pal-E7) at tail base, and the RhB^+^ cells were detected 24 h after injection. The frequencies (**h**) and MFI (**i**) of RhB^+^ DCs and macrophages in DLNs were analyzed. Data are shown as mean±s.d. (*n*=4). *P<0.05; **P<0.01; ***P<0.001; ****P<0.0001. DC, dendritic cell; DLN, draining lymph node; MFI, mean fluorescent intensity; NS, not significant; PEG-PE, polyethylene glycol-phosphatidylethanolamine; RhB, Rhodamine B.

Next, we investigated whether PEG-PE micelles could indeed efficiently target LN APCs and prolong their retention inside LNs. Different FITC-labeled formulations (free FITC, referred as ‘FITC’; FITC-labeled liposome, referred as ‘F-L’ and FITC-labeled PEG-PE micelle, referred as ‘F-M’) were injected into C57BL/6 mice subcutaneously at the tail base. The lymphatic trafficking of the formulations was monitored using an *in vivo* fluorescent image system (IVIS). The images showed that FITC and F-L mostly stayed and largely retained around the injection sites even 7 days post injection, whereas F-M diffused rapidly from the injection site (Supplementary Figure S4A and B) and drained within the interstitial fluid and lymph into the draining LNs (DLNs) within 1 h (Figure 4a), and more importantly accumulated over time and retained over a period of 96 h, much longer than the other formulations (Figure 4a–c). Therefore, the PEG-PE micelle demonstrates efficient and prolonged delivery of payloads to DLNs.

Furthermore, to test whether APCs in DLNs are efficiently targeted by PEG-PE micelle, FITC intensity in DLN cells was measured by flow cytometry. Compared with free FITC, F-M was quickly and efficiently captured by DCs and macrophages both quantitatively (Figure 4d and e) and qualitatively (Figure 4f). At 24 h post injection, about 90% of both migratory DCs (MHCII^hi^) and resident DCs (MHCII^lo^), and 71% macrophages have uptaken significant amount of PEG-PE micelle. Free FITC mixed with PEG-PE micelle (F+M) did not improve the FITC targeting, suggesting the necessity of PEG-PE micellization (Figure 4d–f). In contrast to the efficient uptake of PEG-PE micelles by APCs, lymphocytes are much less targeted (Supplementary Figure S5A–C). Further analysis showed that PEG-PE micelle significantly increased the E7 peptide antigen delivery to LN APCs (Figure 4g–i), and M-MPLA could trigger higher levels of costimulatory molecules on LN APCs (Supplementary Figure S6). Hence, the PEG-PE micelle appears as an appropriate carrier to deliver their payloads to LN APCs for therapeutic vaccine purpose.

### PEG-PE micelle vaccine elicits potent therapeutic antitumor cytotoxic CD8^+^ T-cell response

**Figure 5:**
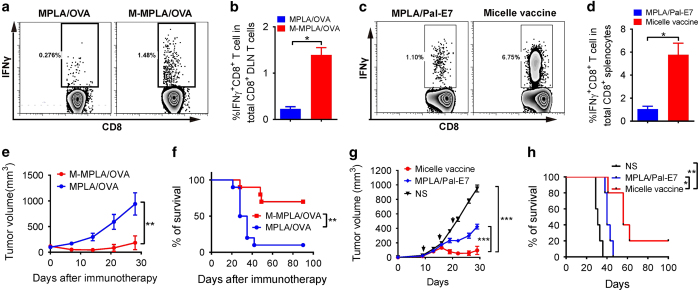
PEG-PE micelle vaccine elicits potent antigen-specific antitumor cytotoxic CD8^+^ T-cell response. (**a** and **b**) DLNs from C57BL/6 mice, which were immunized with OVA micelle vaccine or control vaccine, were isolated and stimulated with SIINFEKL peptide (5 μg ml^−1^) for 6 h in the presence with brefeldin A. The frequencies of IFNγ^+^ cells among total CD8^+^ T cells in DLNs were assessed. The statistical results are shown in **b**. Mean±s.e.m. (*n*=3). Data are representative of three independent experiments. (**c **and **d**) C57BL/6 mice were subcutaneously immunized three times with different vaccine formulations (MPLA mixed with Pal-E7 or the PEG-PE micelle vaccine encapsulating both MPLA and Pal-E7) containing 5 μg Pal-E7 and 2.5 μg MPLA per mouse. Five days after final vaccination, cells isolated from the spleens were stimulated with E7_49-57_ peptide (5 μg ml^−1^) for 6 h. The frequencies of IFNγ^+^ cells among total CD8^+^ T cells in spleen were analyzed. The statistical results are shown in **d**. Mean±s.e.m. (*n*=3). (**e **and **f**) C57BL/6 mice were subcutaneously inoculated with 1×10^6^ MC38-OVA cells at right flank. 12, 19 and 26 days later, tumor-bearing mice were immunized with MPLA/OVA or micelle vaccine (M-MPLA/OVA). The tumor growth curve and the survival curve are shown in **e** and **f**. Mean±s.e.m. (*n*=10). (**g** and **h**) C57BL/6 mice were subcutaneously inoculated with 5×10^4^ TC-1 cells at the right flank. 9, 15 and 20 days later, tumor-bearing mice were immunized with MPLA/Pal-E7 or micelle vaccine. The tumor growth curve and the survival curve are shown in **g** and **h**. Mean±s.e.m. (*n*=5). *P<0.05; **P<0.01; ***P<0.001. DLN, draining lymph node; MPLA, monophosphoryl lipid A; NS, not significant; PEG-PE, polyethylene glycol-phosphatidylethanolamine.

We next further investigated whether the outstanding immunological features of the PEG-PE micelle-based vaccine could be translated into potent T-cell response for tumor control PEG-PE micelle vaccine with OVA peptide was used to immunize the C57BL/6 mice. OVA-specific CD8^+^ T-cell response was measured 7 days after a single immunization. Results showed that a significantly higher CTL response was elicited by the micelle vaccine compared with non-micelle control vaccine (MPLA/OVA; Figure 5a and b). Micellar antigen (M-OVA) alone, without MPLA, generated barely detectable CTL response (data not shown). In addition, HPV16 E7 peptide was also tested. Upon three vaccinations, significantly enhanced E7-specific CTL response was also determined in the micelle vaccine group (Figure 5c and d). Moreover, the vaccinated mice were re-stimulated with MPLA/Pal-E7 90 days after the last vaccination. Results showed that micelle vaccine group had significantly higher number of antigen-specific IFN-γ producing CTL (Supplementary Figure S7). These data confirm that the micelle-based vaccine can increase cross-priming and induce memory for CTLs *in vivo*.

To test the therapeutic effect of the micelle vaccines, we first used the MC38-OVA tumor model. Consistent with the dramatically enhanced OVA-specific CTL response, the tumor growth was efficiently controlled in the micelle vaccination group. The mouse survival rate was also dramatically increased in the micelle vaccination group (70%) compared with non-micelle vaccination (10%; Figure 5e and f). We further tested the antitumor efficacy using true tumor antigen/tumor models: E7 oncoprotein for TC-1 and Trp2 for B16F10. The tumor-bearing mice were generated and vaccinated as depicted. Encouragingly, in the TC-1 model, the non-micelle MPLA/Pal-E7 vaccine could only modestly inhibit the tumor growth and extend the mice survival, while the micelle vaccine led to sustained regression of TC-1 tumors and significantly improved the survival of the tumor-bearing mice (Figure 5g and h). Similar antitumor response was found in B16F10 model when M-MPLA/Trp2 micelle vaccine was used (Supplementary Figure S8A and B). Thus, PEG-PE micelle may be a useful platform for different therapeutic tumor vaccines.

To determine whether the antitumor effect of micelle-based vaccine is dependent on CTL response, tumor-bearing mice were depleted of CD8^+^ T cells systemically by intraperitoneal injection of depleting antibody during vaccination. The results showed that depletion of CD8^+^ T cells largely abolished the antitumor effects of micelle-based vaccine immunotherapy (Supplementary Figure S9) indicating an essential role of CTL in the micelle vaccine induced therapeutic effect. Overall CD4^+^ T-cell depletion did not result in impaired antitumor effect of the micelle vaccine (Supplementary Figure S9). However, it should be noted that CD4 depleting antibody used here depletes both CD4^+^ T-helper cells and Treg cells. Further studies, such as specific Treg depletion, are required to clarify their specific roles.

### Combination of micelle vaccine immunotherapy with chemotherapy or surgical operation produces more potent antitumor effect

**Figure 6:**
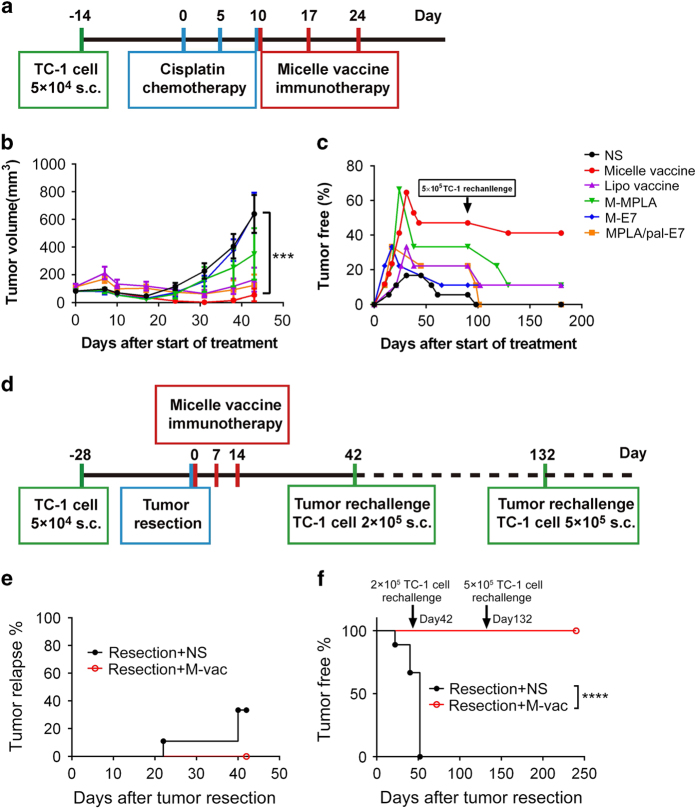
Micelle vaccine synergizes with chemotherapy or surgical operation for enhanced tumor control. Schematic outline of the experiment designs for combinational therapy of micelle vaccine with either chemotherapy (**a**) or surgery (**d**). ( **b** and **c**) The tumor growth curve and tumor-free percentage (100 days after chemotherapy) of micelle vaccine and control vaccines with chemotherapy (*n*=10). At the same day of surgery, the mice were immunized with micelle vaccine (red line), normal saline (NS, black line) or other controls, then boosted twice at 7 days interval. (**e**) Tumor relapse curve was shown (*n*=9). Subsequently, the tumor-free mice were re-challenged with 2×10^5^ and 5×10^5^ TC-1 cells at day 42 and day 132, respectively. (**f**) The tumor-free percentage was shown (*n*=6 for NS control; *n*=9 for micelle vaccine). ***P<0.001; ****P<0.0001. MPLA, monophosphoryl lipid A.

Chemoresistance and tumor relapse are usually associated with dysfunctional CTL response. Proper cytotoxic CD8^+^ T-cell responses have been found critical for eradication of tumor after chemotherapy [45]. Given the strong CTL response elicited by PEG-PE vaccines, we wondered whether combinational therapy would help to overcome chemoresistance and preventing tumor relapse. Therefore, we tested the efficacy of the micelle vaccine in combination with regular chemotherapy. Mice bearing the established TC-1 tumors were treated as depicted (Figure 6a). The combination of cisplatin and the micelle vaccine treatment potently suppressed tumor growth (Figure 6b), which continued for at least 8 weeks from the start of the treatment, and with undetected systemic toxicity represented by body weight loss (Supplementary Figure S10). More strikingly, more than 50% mice remain tumor-free at 3 months after combinational treatment, even with high dose of tumor re-challenge, whereas none is tumor-free in the chemotherapy single treatment group (Figure 6c). It suggests that additional vaccine is essential to reduce tumor relapse post chemotherapy.

Surgery is usually the primary clinical choice for most solid tumors. However, resection alone is rarely curative for advanced tumors due to either local tumor recurrence or outgrowth of micrometastasis. To test whether micelle vaccine could be effective for tumor recurrence control after surgery, established TC-1 tumors were first resected and then the mice were immunized with micelle vaccines or saline control (Figure 6d). Forty-two days later, tumor relapse occurred in three of nine mice in the control group, whereas none in the micelle vaccine group (Figure 6e). To explore whether combinational therapy could promote prolonged antitumor immune response, remaining tumor-free mice were re-challenged 6 weeks after surgery. All the mice in the unimmunized group relapsed with tumor; in stark contrast, all the mice in the combinational treatment group were still tumor-free, even upon a secondary challenge with high dose of tumor cells (Figure 6f). These data suggest the potency of the micelle vaccine on elicitation of long-term protective immunity in combination with conventional treatments.

## Discussion

Various technical challenges hamper the development of efficient therapeutic tumor vaccines for tumor control. Several factors are favorable for an ideal therapeutic, CTL-eliciting tumor vaccine: (i) cytosolic antigen delivery, which favors the processing and cross-presentation of exogenous antigens in MHC-I pathway; (ii) synchronously adequate DC activation for antigen presentation and co-stimulation of CD8^+^ T cells; (iii) quantitatively sufficient number of DCs are used; and (iv) spatially are these DCs in LNs. To our knowledge, the current PEG-PE micelle system has fulfilled all the above demands in one formulation.

Cytosolic antigen delivery has long been pursued for the development of therapeutic vaccines. CPP has been widely studied for this purpose. However, the fusion of CPP may alter the subcellular localization of the antigen and impair its processing pathway [9–11]. In current study, we have found PEG-PE micelles is an efficient carrier for cytosolic delivery of antigen peptide. Different from CPP, it delivers peptides to the cell membrane, but does not accompany them into the cytosol (Figure 1f), thus avoiding any intracellular influence of the material on the target cells. More interestingly, PEG-PE micelles can transform peptides from non-α-helix into α-helix, which is favorable for membrane translocation [46]. In fact, several endeavors have been made to stabilize peptides in α-helix for better cytosolic delivery [12–16]. In the current study, we have provided an easier approach for this by PEG-PE micelle encapsulation. How PEG-PE micelles enable α-helix transformation is unclear. One possibility is that PEG-PE micelles provide an electrostatic environment for peptides for conformation modulation and stabilization. In fact, previous studies have showed that micellar surface charge can render random coiled folding in aqueous solution into α-helical conformation in micelles via electrostatic interactions [47, 48]. If so, it should be noted that the electrostatic feature of peptides may be also important for its α-helix transformation in PEG-PE micelles. In fact, not all peptides can be transformed to α-helix by PEG-PE according to our unpublished data. How to improve the cytosolic delivery of these peptides by PEG-PE remains an interesting question in future. In addition, it should be noted that PEG-PE micelle-mediated peptide delivery is a complex process and not simply determined by the peptide conformation. Besides this, other properties of the peptides such as hydrophobicity/hydrophilicity and charge are also important for their intracellular delivery [49, 50]. Furthermore, PEG-PE micelle may influence the interaction between the peptides and cell membrane proteins/lipids, therefore, affects their intracellular delivery [30]. Further studies are required to evaluate the relative contribution of different factors on cytosolic delivery.

MPLA has been proved by FDA and widely used for vaccine adjuvant. However, as a detoxic form of LPS, the adjuvant activity is also attenuated. In our study, we found PEG-PE micelles enhance MPLA activity about 100-fold more as demonstrated by production of TNF-α, IL-6 and IL-12. The constimulatory molecules were also markedly enhanced by micellized MPLA compared with non-micellized MPLA. One reason of PEG-PE micelles accounting for these may be its monomerization of MPLA. MPLA, as well as LPS, has very low critical micelle concentration (~10^−9^ m), thus are usually present as aggregates [32, 33]. Therefore, LBP is required for transport single LPS/MPLA molecule to TLR4/MD-2 with the help of CD14. Without LBP, LPS/MPLA activity is dramatically reduced. However, PEG-PE micellized MPLA is monomerized during preparation, thus favorable for binding with its receptors. Besides, the character of membrane fusion of PEG-PE micelles may further assist MPLA-TLR4–MD-2 complex formation for rapid signaling [39]. Therefore, our data strongly demonstrated that PEG-PE micelle serving as an artificial molecular ‘chaperon’ greatly amplified the adjuvant activity of MPLA through dispersing MPLA into the form of monomer in the micelle, and then efficiently transferring MPLA to cell membrane for receptor binding by membrane insertion.

Quantitatively sufficient number of DCs is also required for T-cell priming in LNs. A lot of attempts have been made for this purpose. Recently, a delicate injectable scaffold system was created allowing attraction of massive immune cells (especially DCs) into the microenvironment for DC modulation [51]. However, the efficient migration of DCs to the DLNs may still be a limiting factor, as T cells ideally need to be primed in the LNs. In fact, increased DC migration to DLNs has been shown correlated with enhanced tumor control by DC vaccines [52]. On the other hand, given the presence of abundant DCs in LNs, LN DC targeting has become a popular way. Taking the advantage of the lymphatic targeting character of albumin, a recent study demonstrated highly efficient LN targeting effect of an amphiphilic vaccine [25]. In the current study, PEG-PE micelles also showed outstanding LN targeting efficiency in DLNs upon subcutaneous administration. Different from albumin-mediated LN targeting, this may be due to the proper size of PEG-PE micelles for lymphatic trafficking [53]. In addition, different from the amphiphilic vaccine, PEG-PE micelle vaccine allows co-delivery of both antigen and MPLA adjuvant to the same target APC. In fact, individual encapsulation and delivery by PEG-PE micelles significantly reduced the induction of CTL response. Furthermore, PEG-PE micelle allows cytosolic antigen delivery and amplification of MPLA activity. Thus, PEG-PE micelle-based vaccine appears to combine multiple pursued features of a therapeutic vaccine in one formulation and becomes a unique platform for the development of therapeutic vaccines.

Tumor immunotherapy has been increasingly appreciated in recent years, especially when in combination with traditional approaches. Given the prominent effect of our current micelle vaccine on induction of cytotoxic T-cell response, we tested its therapeutic efficacy in several aggressive solid tumor models. Combination with conventional chemotherapy demonstrated more notable efficacy on primary tumor control, even when administrated 24 days after tumor implantation when tumor has been well established. Combination with surgery, a striking long period of protection was found against spontaneous tumor recurrence, even upon active high-load tumor cell re-challenge. Therefore, the PEG-PE micelle vaccine has demonstrated encouraging efficacy for both tumor control and memory protection and harbors a great potential for clinical tumor treatment.

## Materials and Methods

### Mice

Female C57BL/6 mice (6–8 weeks old) were purchased from Vital River Laboratory Animal Technology Co. (Beijing, China). OVA_257-264_-specific TCR transgenic mice C57BL/6-Tg (TcraTcrb)1100Mjb/J(OT1) were purchased from Jackson Laboratories (Bar Harbor, ME, USA). All the mice were housed under pathogen-free conditions in the animal care facilities at the Institute of Biophysics, Chinese Academy of Sciences. All animal experiments were approved by the Institutional Laboratory Animal Care and Use Committee at the Institute of Biophysics, Chinese Academy of Sciences.

### Preparation of PEG-PE micelle vaccines

The micelle vaccine was prepared by film-rehydration method as previously described [27]. Briefly, MPLA (Avanti Polar lipids, Alabaster, AL, USA) was dissolved in chloroform/methanol (2:1). Pal-E7 (HPV16 E7_43-62_, GQAEPDRAHYNIVTFCCKCD, N terminus with palmitic acid modification), OVA peptide (OVA_250-264_, SGLEQLESIINFEKL) or TRP2 peptide (TRP2_180-188_, SVYDFFVWL) were dissolved in methanol, respectively. Then mixed 200 μg peptides and 100 μg MPLA with 10 mg PEG-PE (Lipoid, Newark, NJ, USA), which dissolved in chloroform (Sigma, St Louis, MO, USA) in a molar ratio of about 180:4:3 (PEG-PE: peptide: MPLA). The organic solvents were removed using a rotary evaporator to form the antigen peptide-containing lipid film. To improve the assembly efficiency, the lipid film was hydrated with 1 ml sterile deionized H_2_O at 53±1 °C for 30 min under the protection of nitrogen. All the peptides in this study were synthesized by GL Biochem (Shanghai, China).

### *In vitro* imaging analysis

DC2.4 cells or EGFP-Rab5 expressing DC2.4 cells were seeded in 35 mm glass-bottom plates. After overnight culture, different formulations of indicated Rhodamine-labeled peptides (20 μm) were added and incubated at 37 °C for 30 min or 2 h. Then, 100 nm Lyso-Tracker Green or ER-Tracker Green (Life Technologies, Grand Island, NY, USA) was added and incubated for 5 or 20 min, respectively. The processes of PEG-PE inserting into cell membranes, peptide internalization and the co-localization of peptides with early endosome (Rab5), lysosome or ER were imaged using a confocal microscope LSM-700 (ZEISS, Germany).

### *In vivo* imaging analysis

C57BL/6 mice were subcutaneously injected with 15 μmol (FITC) of different FITC-labeled formulations (FITC, F-L, F-M) at tail base. The diffusion from injection sites was detected using an *in vivo* imaging system (IVIS) (Caliper life Sciences, Hopkinton, MA, USA). Spectrum unmixing technique was applied to reducing the effect of native tissue auto-fluorescence, separating detecting fluorescence and analyzing fluorescence diffusion. For imaging on inguinal and axillary LNs, LNs were excised and imaged at indicated time points. An image set (Ex: 500, Em: 540, f4, bin 8, 2 s) was collected. Living Image software Version 4.4 (Caliper Life Sciences, Waltham, MA, USA) was used to acquire and quantify the fluorescence imaging data sets.

### Flow cytometry and antibodies

Single-cell suspensions were processed and 2×10^6^ cells per sample were used to perform flow cytometry assay. First, the cells were incubated with an anti-FcγR mAb (2.4G2) to block nonspecific binding. Then, fluorescence-labeled antibodies were added with specific purpose. DCs were stained with anti-CD11c (N418) and anti-MHCII (M5/114.15.2) antibodies, and macrophages were stained with anti-CD11b (M1/70) and anti-F4/80 (BM8) antibodies. For phenotypic maturation assay, anti-CD40 (1C10), anti-CD80 (16-10A1) and anti-CD86 (GL1) antibodies were used. For intracellular staining, the cell suspensions were re-stimulated in U-bottom 96-well plates with 5 μg ml^−1^ corresponding peptides for 6 h in the presence of Brefeldin A (5 μg ml^−1^). After blocking with an anti-FcγR mAb (2.4G2), the cells were stained with antibodies against CD8 (53-6.7) before fixation/permeabilization and intracellular staining for IFNγ (XMG1.2). Other antibodies used in this study were described in related methods. Finally, the samples were detected by BD FACSCalibur or BD LSRFortessa flow cytometer (BD Biosciences, San Jose, CA, USA) and the data were analyzed using the FlowJo software (Tree Star Inc., Ashland, OR, USA). All the antibodies were purchased from eBioscience (San Diego, CA, USA) or Tonbo Biosciences (San Diego, CA, USA).

### Tumor models and vaccination

C57BL/6 mice were subcutaneously inoculated with 5×10^4^ TC-1 cells, 1×10^6^ MC38-OVA cells at the right flank. After the tumor was established, the mice were immunized with different vaccine formulations (non-micelle vaccine: free MPLA mixed with peptide, pal-E7, OVA; micelle vaccine: the micelle encapsulating both MPLA and peptides) every week. The mice were executed once the tumor size researched or exceeded 1 000 mm^3^, in accordance with established guidelines.

### Statistical analysis

Statistical analysis was performed using GraphPad Prism statistical software (GraphPad Software Inc., San Diego, CA, USA). Tumor growth and survival curves were analyzed by two-way analysis of variance or log-rank (Mantel–Cox) tests, respectively. All other data were analyzed using unpaired two-tailed *t*-tests. A value of *P*<0.05 was considered statistically significant (*P<0.05; **P<0.01; and ***P<0.001; ****P<0.0001).
