# Supplementary material for: Coordinating antigen cytosolic delivery and danger signaling to program potent cross-priming by micelle-based nanovaccine
Source: Cell Discov. 2017 Apr 4;3:17007–. doi: 10.1038/celldisc.2017.7 (PMC5379745; doi:10.1038/celldisc.2017.7)
Supplement: Supplementary information [file celldisc20177-s1.pdf]

Coordinating Antigen Cytosolic Delivery and Danger Signaling to Program Potent Cross Priming by Micelle-based Nanovaccine

Zhida Liu[1,3,4,5], Chang Zhou[2,3,5], Yan Qin[2,5], Zihao Wang[2,3], Luyao Wang[2,3], Xiuli Wei[2], Yinjian Zhou[2], Qicheng Li[2], Hang Zhou[1,3], Wenjun Wang[1,3], Yang-Xin Fu[1,4*], Mingzhao Zhu[1,3*] & Wei Liang[2,3*]

[1] Key Laboratory of Infection and Immunity, Institute of Biophysics, Chinese Academy of Sciences, Beijing 100101, China.

[2]Protein & Peptide Pharmaceutical Laboratory, Institute of Biophysics, Chinese Academy of Sciences, Beijing 100101, China.

[3] University of Chinese Academy of Sciences, Beijing 100049, China.

[4] The Department of Pathology and Immunology, UT Southwestern Medical Center, Dallas, TX 75235-9072, USA.

[5]These authors contributed equally to this work.

[*]Correspondence should be addressed to: Wei Liang (weixx@sun5.ibp.ac.cn) or Mingzhao Zhu (zhumz@ibp.ac.cn) or Yang-Xin Fu (Yang-Xin.Fu@UTSouthwestern.edu)

Supplementary Materials

Figure S1

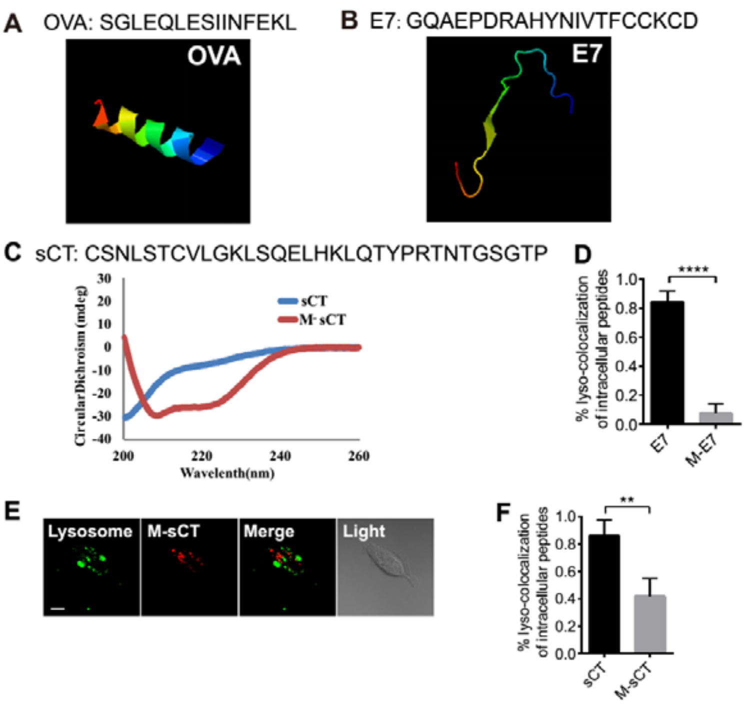

Figure S1. PEG-PE nanomicelle efficiently delivers the antigens into the cytosol. The sequence and the most possible structural conformations of OVA peptide (**A**) and E7 peptide (**B**). (**C**)The secondary structure of sCT peptide (sCT) and PEG-PE micellized sCT (M-sCT) was determined by CD spectroscopy. (**E**) DC2.4 cells were incubated with 20 μM PEG-PE micellized RhB-labeled sCT peptide for 30min. Subsequently, LysoTrackerGreen was used to track lysosomes, and then the co-localization of intracellular RhB-peptide and lysosomes were analyzed by confocal scanning microscope. Scale bar =10μm. (**D, F**) Quantification of the intracellular distribution of antigenic peptides. 20 μM RhB labeled E7 (**D**) or sCT (**F**) peptides in the free form or in PEG-PE micelles were incubated with DC2.4 cells for 30min. Cells were fixed and the intracellular distribution of peptides were determined by confocal imaging. LysoTrackerGreen was used to track lysosomes. The graphs show

the percentage of the lysosome-colocalized peptide area. The colocalization area was analyzed by ImageJ (FIJI) coloc 2 (n>30 cells/group)[1].

Figure S2

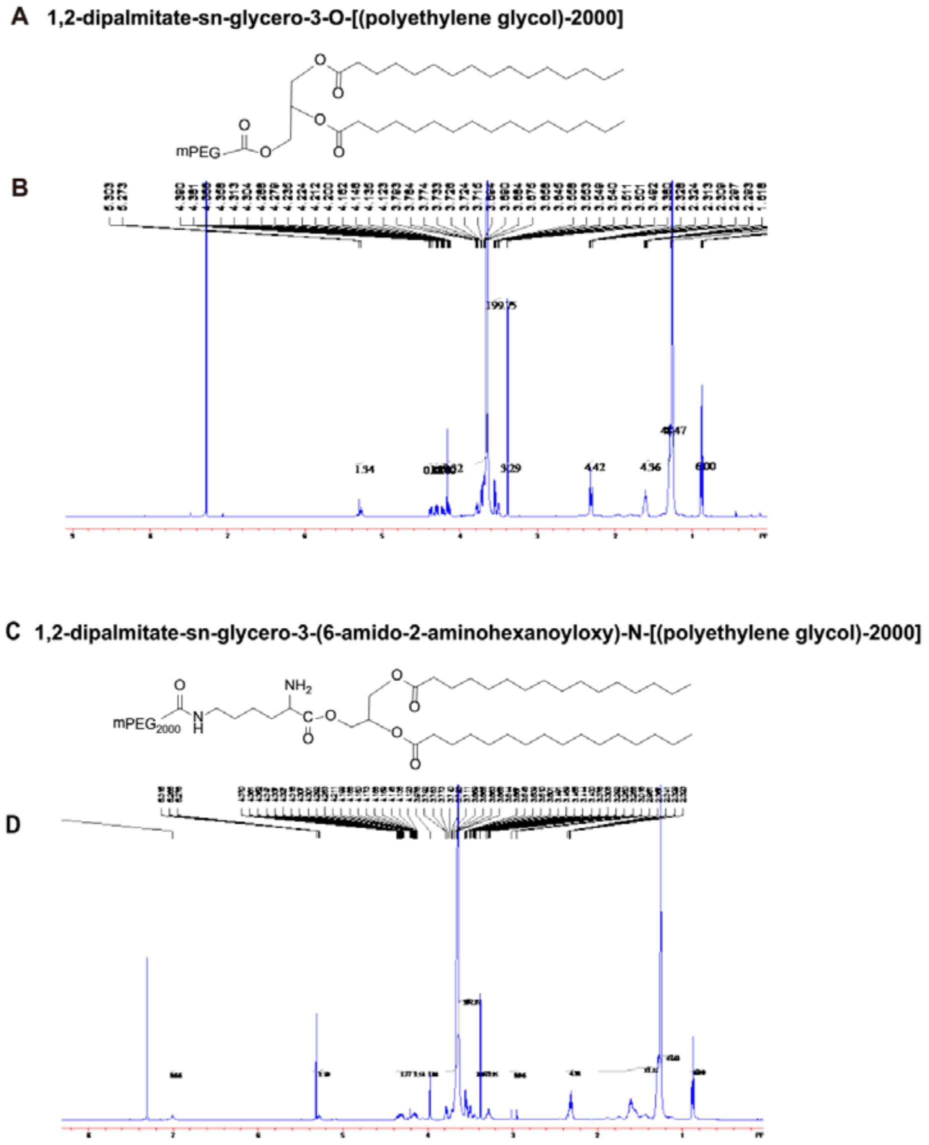

Figure S2. Chemical structure of PEG_{2000}-DPG and PEG_{2000}-L-DPG. Chemical structure of PEG_{2000}-DPG (**A**) and PEG_{2000}-L-DPG (**B**). ^{1}H NMR spectra of PEG_{2000}-DPG (**C**), and (**D**) PEG_{2000}-L-DPG.

Figure S3

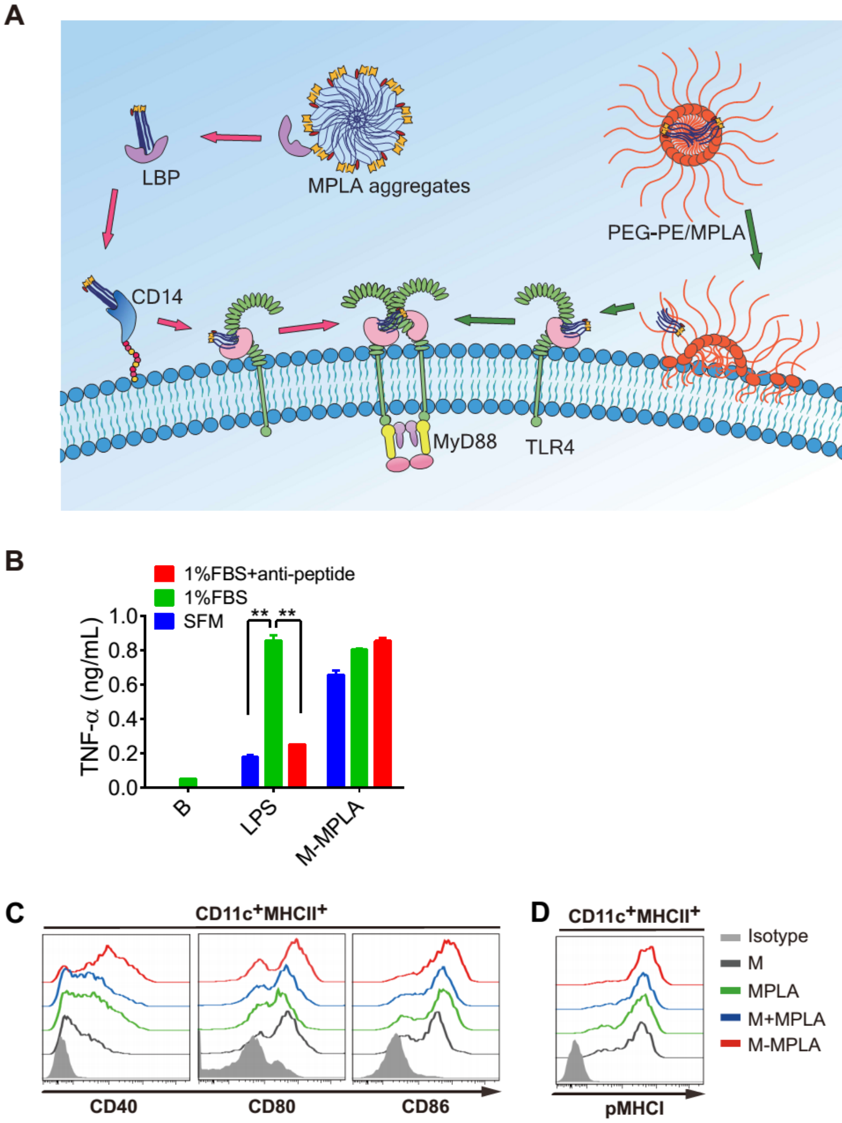

Figure S3. PEG-PE micelle chaperons MPLA for enhanced TLR signaling and APC function. (**A**) Schematic diagram of TLR4 signaling activation by M-MPLA (PEG-PE/MPLA). (**B**) RAW 264.7 cells were treated with 100ng/ml LPS, M-MPLA in the presence and absence of 100μg/ml LBP-blocking peptide (LBPK95A) for 2h. Then the secreted TNF-α was determined by ELISA. Mean ± SD (n=3). **, P<0.01. (**C**) Histogram of Fig. 2G. Splenocytes from C57BL/6 mice were incubated with different formulations of MPLA (100ng/ml) for 12h. Then the $CD11c^+$ $MHCII^+$ DCs were gated for CD40, CD80 and CD86 detection by FACS. (**D**) Histogram of Fig.2H.

Splenocytes from C57BL/6 mice were incubated with 100ng/ml different formulations of MPLA in the presence of 7μM Pal-OVA for 16h. Then, the complex of MHCI and peptide (SIINFEKL) (pMHCI) was detected by flow cytometry.

Figure S4

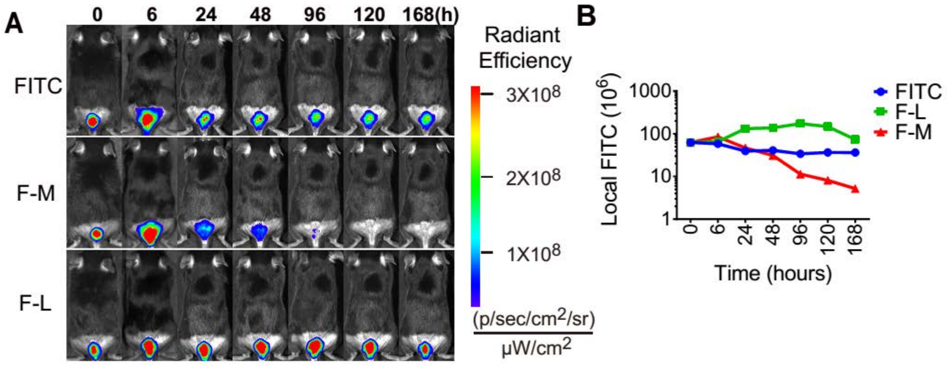

Figure S4. PEG-PE micelle diffused slowly and largely from injection site.(**A**) C57BL/6 mice were s.c. injected with 15μmol/mouse different FITC formulations (FITC, FITC labeled PEG-PE micelle, FITC labeled liposome) at tail base. Then the diffusion from injection sites of different FITC formulations were monitored by IVIS fluorescence imaging at indicated time points (from 0h to 168h). (**B**) The fluorescent intensity was measured and quantified using the Living Image 4.4 software.

Figure S5

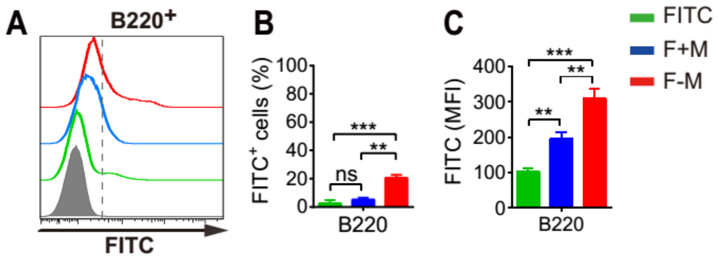

Figure S5. PEG-PE micelle rarely accumulated in B cells in DLN. (**A**) The B cells in DLNs were determined by flow cytometry at 24h post-injection. The frequencies (**B**) and mean fluorescence intensity (MFI) of the $FITC^{+}$ cells (**C**) were analyzed respectively. The statistical results are shown as Mean ± SD (n=4). *, P<0.05; **, P<0.01; ***, P<0.001; ns, not significant.

Figure S6

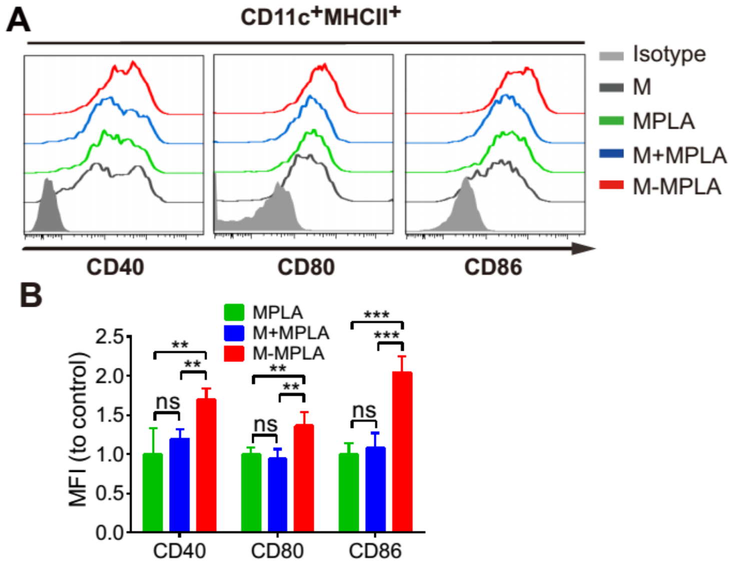

Figure S6. PEG-PE micellization enhanced DLN APCs activation by MPLA. (**A**) C57BL/6 mice were s.c. injected with different formulations of MPLA (MPLA 2.5μg/mouse) at tail base. 24h later, the DLNs were digested and CD11c^{+} MHCII^{+} DCs were gated for CD40, CD80 and CD86 detection. The expression level of CD40, CD80 and CD86 on DCs were analyzed. The statistical results are shown in panel (**B**). Data are shown as Mean ± SD (n=4). **, P<0.001; ***, P<0.01; ns, not significant.

Figure S7

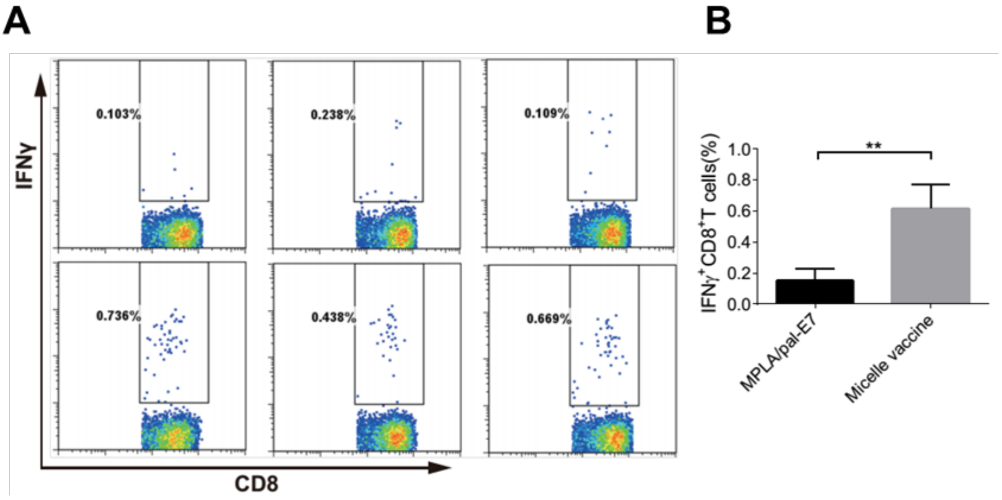

Figure S7. Micelle vaccine generated better antigen-specific memory CTL response comparing to non-micelle vaccine. (A) C57BL/6 mice were subcutaneously immunized three times with different vaccine formulations (MPLA mixed with Pal-E7 or the PEG-PE micelle vaccine encapsulating both MPLA and Pal-E7) containing 5μg Pal-E7 and 2.5μg MPLA per mouse. 90 days later, all mice were reimmunized with MPLA/Pal-E7. After 5 days, cells isolated from the spleens were stimulated with $E7_{49\text{-}57}$ peptide (5μg/ml) for 6h. The frequencies of $IFN\gamma^{+}$ cells among total $CD8^{+}$ T cells in spleen were analyzed. The statistical results are shown in panel B. Mean ± SD (n=3). **, P<0.01.

Figure S8

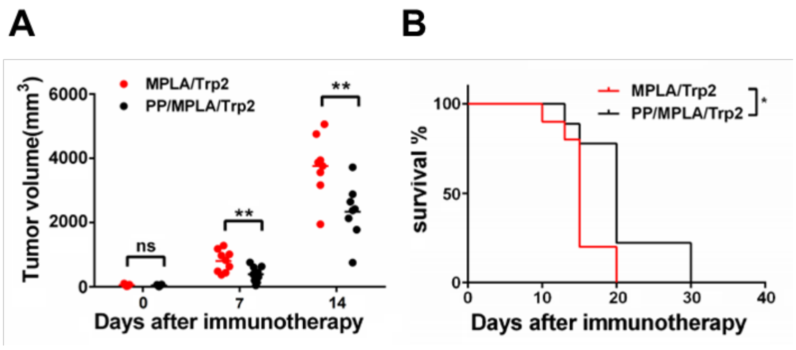

Figure S8. Micelle vaccine demonstrates better tumor control than non-micelle vaccine on B16F10 tumor model. C57BL/6 mice were inoculated with 105 B16F10 cells at right flank. Then, tumor bearing mice were immunized with MPLA/Trp2 or micelle vaccine PP/MPLA/Trp2 (TRP2, 10μg/mouse; MPLA, 2.5μg/mouse) at day 7, 14, and 21. Tumor size was measured every week (A). The survival curves were shown in panel B. (n=9). *, P<0.05; **, P<0.01.

Figure S9

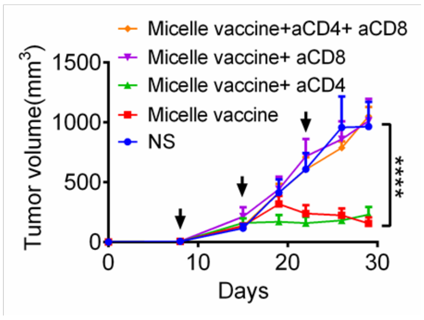

Figure S9. Micelle vaccine elicits potent anti-tumor efficiency mostly dependent on cytotoxic CD8+ T cell response. C57BL/6 mice were s.c. inoculated with 5×104 TC-1 cells at right flank and treated with micelle vaccine (5μg Pal-E7 and 2.5μg MPLA per mouse) on day 8, 15, 22. Additionally, 200μg of a CD4-depleting antibody (GK1.5) or CD8-depleting antibody (TIB210) was administered intraperitoneally on day 8, 15, 22. Mean ± SEM (n=5). ****, P< 0.0001.

Figure S10

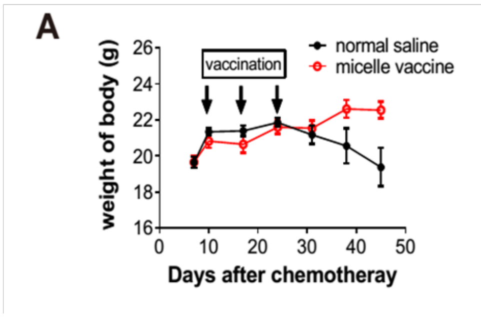

Figure S10. Body weight loss of micelle vaccine treated mice after chemotherapy. Corresponding to Fig. 6A, body weights of micelle vaccine & cisplatin combination treated mice were monitored (n=10). The treatment strategies were described in method.

Supplementary Methods

Cell lines

TC-1, a tumor cell line transformed from C57BL/6 primary mouse lung cells, was kindly provided by Dr. Xuemei Xu (Institute of Basic Medical Sciences Chinese Academy of Medical Sciences & School of Basic Medicine, Peking Union Medical College, Beijing, China). DC2.4, an immortalized DC cell line derived from C57BL/6 mice, was kindly provided by Dr. Li Tang (Key Laboratory of Protein Engineering, Academy of Military Medical Sciences, Beijing, China). RAW 264.7, a macrophage like cell line derived from BALB/c mice, was purchased from the Type Culture Collection of the Chinese Academy of Sciences (Shanghai, China). MC38 is a murine colon adenocarcinoma cell line. MC38-OVA was sorted and subcloned after MC38 cells were stable transduced with retrovirus expressing mouse EGFRVIII–OTI. B16-F10 murine melanoma cell line was originally obtained from ATCC, catalog no. CRL-6475. All cells were cultured in complete RPMI1640 medium according to ATCC recommendations.

Preparation of PEG-PE micellized antigenic peptides

Briefly, peptides (HPV16 $E7_{43\text{-}62}$, $OVA_{250\text{-}264}$, sCT) or Rhodamine labeled peptides (RhB-peptide) were dissolved in methanol, respectively. Then the three peptides were respectively mixed with corresponding amount PEG_{2000}-PE or FITC labeled PEG_{2000}-PE(FITC-PP) which was dissolved in chloroform in a 1/10 molar ratio of peptide/PEG-PE. Then the peptide containing micelle were prepared as described in

the **Preparation of PEG-PE micelle vaccines**.

Secondary structure analysis of the peptides by Circular dichroism (CD) spectroscope

Secondary structure of the peptides was determined by CD spectroscopy. 100μg/ml peptides or PEG-PE loaded peptides (in 1/10 molar ratio of peptide/PEG-PE) were scanned at room temperature in a 1-mm path length fused quartz cuvette using a spectropolarimeter (Applied Photophysics Ltd, UK). Spectra were obtained from 200 to 260 nm at 1-nm bandwidth, 5-nm step and 1s response time averaged over 6 runs.

Transmission electron microscopy

The size and morphology of micelle vaccine were analyzed by negative staining and transmission electron microscopy (TEM). Micelle vaccine or empty micelles were diluted to a concentration of 1 mg/ml PEG-PE with deionized water. The micelles were stained with 1% uranyl acetate and examined with a Tecnai spirit TEM (FEI, Hillsboro, OR).

Dynamic light scattering (DLS)

To determine the particle size distribution of the micelle vaccine or empty micelles, dynamic light scattering (DLS) measurement was performed using a Zetasizer Nano ZS (Malvern Instrument, Malvern, Worcestershire, U.K.) at 633 nm and a 173º scattering angle at 25°C. The micelles were diluted to the PEG-PE concentration of

0.5 mg/ml with deionized water. Particle sizes were shown as intensity-weighted diameters. The values were reported as the mean ± standard deviation (SD) based on more than three individual measurements performed in triplicate.

MPLA activity assay

4×10^5 RAW264.7 cells were seeded in 24-well plates overnight. Then, the cells were treated with 10, 100, 1000ng/ml MPLA, M-MPLA, Lipo/MPLA, PLA/MPLA, or PEG_{2000}-DPG (1,2-dipalmitate-sn-glycero-3-O-[(polyethylene glycol)-2000]) micellized MPLA (DP-M-MPLA), PEG_{2000}-L-DPG (1,2-dipalmitate-sn-glycero-3-(6-amido-2-aminohexanoyloxy)-N-[(polyethylene glycol)-2000]) micellized MPLA (N-M-MPLA), respectively for 2 h prior to harvesting the supernatant. TLR4 activation was measured by the cumulative production of TNF-α using the mouse TNF-α ELISA kit (eBioscience, San Diego, CA, USA).

M-MPLA activity assay in serum free medium or under LBP-blocking condition

RAW264.7 cells were seeded and cultured overnight. Then the cells were either treated (1) with serum-free medium (SFM) or (2) with LBP-blocking peptide. (1): The cells were washed with SFM three times before experiment, then treated with 100ng/ml MPLA, M-MPLA, or LPS in the presence or absence of serum respectively; (2):The cells were treated with or without LBPK95A (LBPK95A sequence: RVQGRWKVRKSFFK) in 1% FBS for 4h. Then, 100ng/ml LPS or M-MPLA was added and incubated for 2h prior to harvesting the supernatant. TLR4 activation was

measured as described above.

TLR4/MD-2 heterotetramer formation detection

RAW264.7 cells were stimulated with 100ng/ml MPLA, M+MPLA, M-MPLA, or LPS for 15min at 37℃. Then the treated cells were put on ice and washed with PBS. After blocking with anti-FcγR mAb (2.4G2), the cells were incubated with one of the following antibodies: MTS510 PE, Rat IgG2a K isotype control PE. Finally, the cells were analyzed by flow cytometry (Calibur, BD Bioscience).

Bone marrow dendritic cells (BMDC) generation

BMDCs are generated as described previously [2]. Briefly, bone marrow (BM) cells were collected from tibias and femurs of female C57BL/6 mice. At day 0, 2×10^6 BM cells were cultured in 100mm plate with 10ml complete RPMI 1640 medium containing 200 U/ml rmGM-CSF (PeproTech, Rocky Hill, NJ). At day 3, another 10 ml RPMI 1640 medium containing 200U/ml rmGM-CSF were added. At day 6, the immature BMDCs were collected and ready to use.

Cytokines measurement

BMDCs or splenocytes from C57BL/6 mice were seeded in 96-well plates and cultured in the presence of 100 ng/ml different MPLA formulations (MPLA, M+MPLA, M-MPLA), or 10 μg empty PEG-PE micelle. For RNA detection, BMDCs were collected 1h after stimulation. For protein detection, the supernatants were

harvested 24h after stimulation, then the cytokines were measured by ELISA kits (eBioscience, San Diego, CA, USA) using a microplate reader (SpectraMax Plus, Molecular Devices, Sunnyvale, CA, USA).

Real-time quantitative PCR

Total RNA was extracted with RNeasy Mini Kit (QIAGEN, Germany) and cDNA was reverse-transcribed using Revert Aid™ First Strand cDNA Synthesis Kit (Thermo Scientific Fermentas, USA). RT-PCR was performed with SYBR® Green PCR Master Mix (Thermo Scientific Applied Biosystems, USA) using the appropriate primers. Mouse IFNβ (forward primer: CCATCCAAGAGATGCTCCAG, reverse primer: GTGGAGAGCAGTTGAGGACA).

Antigen cross-presentation assay

To detect whether the micellized MPLA can enhance the cross-presentation ability of DCs, 10^7 splenocytes were incubated with 20μg/ml $OVA_{250-264}$ peptide in the presence of different formulations of MPLA (MPLA, M+MPLA or M-MPLA, 100ng/ml MPLA of all formulations) for 16h. Then, the DC subset ($CD11c^+MHCII^+$) in splenocytes was analyzed by flow cytometry using the anti-H2-K^b/SIINFEKL antibody (25-D1.16).

***Ex vivo* cross-priming assay**

The $CD11c^+$ $MHCII^+$ DCs were sorted from splenocytes of naïve C57BL/6 mice by

BD FACS AriaII flow cytometer (BD Biosciences, USA), and pretreated with 5μg/ml $OVA_{250-264}$ peptide in the presence of M+MPLA or M-MPLA (20 ng/ml) for 16h. Then the responding OT1 $CD8^+$ T cells, labeled with CFSE, were added into DCs for co-culture. Briefly, splenocytes (2×10^7 cells/ml) from OT1 transgenic mice were labeled with 5μM CFSE (Invitrogen, Carlsbad, CA, USA) for 10 min, after which 20% FBS was immediately added to terminate the labeling reaction. Then APC-anti-CD8α and PE-anti-TCR Vα2 antibodies were used to sort OT1 CD8 T cells. DCs and T cells were co-cultured at a ratio of 1:3 for 4 days and cultures were prepared in the U-bottom 96-well culture plate in a final volume of 0.2 ml medium containing 5×10^3 sorted DCs and 1.5×10^4 OT1 T cells. Finally, the CFSE dilution on OT1 T cells was detected by flow cytometry and analyzed using FlowJo software (Tree Star, Inc., Ashland, OR, USA). All antibodies were purchased from eBioscience (San Diego, CA, USA).

Preparation of fluorescence labeled PEG-PE micelle and liposome

Synthesis of FITC-PEG-PE was performed as follows. 10 mg (0.0036 mmol) NH_2-PEG_{2000}-DSPE was dissolved in 5 ml chloroform with 0.93 μl DIEA. 1 ml methanol containing 15.6 mg (0.04 mmol) Fluorescein isothiocyanate (FITC, Sigma) was added into the solution prepared above. The solution was stirred under N_2 in dark overnight. After removing solvent under reduced pressure, 0.5 mg (0.0072 mmol) hydroxylamine hydrochloride in 2 ml methanol was added and reacted for 2 h. FITC-PEG-PE was purified by Sephadex-G25 column, and then lyophilized to get the

target product.

FITC labeled PEG-PE micelle (F-M) was prepared as follow. 1mg FITC-PEG-PE and 9 mg PEG-PE were dissolved in chloroform to a final proportion of about 10% FITC-PEG-PE. The organic solvents were removed by a rotary evaporator. The lipid film was hydrated by 1ml physiological saline at 53±1°C for 30min in protection of nitrogen.

FITC labeled liposome (F-L) was prepared by film-rehydration method and extrusion technique. 3.5 mg Cholesterol, 0.73 mg DMPG, 5.8 mg DPPC and 1 mg FITC-PEG-PE were dissolved in chloroform/methanol (2:1). The organic solvent was removed by a rotary evaporator. The lipid film was hydrated by 1ml physiological saline as described previously. Extrusion of FITC-liposome was performed by Mini-Extruder (Avanti Polar Lipids, Alabaster, AL) through a polycarbonate membrane with a pore size of 0.4μm.

***In vivo* LN targeting detection**

To detect the LN targeting of PEG-PE micelle, C57BL/6 mice were subcutaneously injected with different FITC formulations (FITC, F-L, F-M) at tail base, 15μmol/mouse. The DLNs were excised and digested into single cell suspension at 24h and 96h post-injection, $FITC^{+}$DCs and Macrophages were detected by flow cytometry.

To detect the LN targeting delivery of polypeptide antigens by micelle, C57BL/6 mice were subcutaneously injected with 14.6 nmol different formulations of RhB labeled E7 peptide (E7, Pal-E7 and M-E7) at tail base. The DLNs were excised and digested into single cell suspension at 24h post-injection, the RhB^+ DCs and Macrophages cells were detected by flow cytometry.

Vaccination combined with cisplatin chemotherapy

C57BL/6 mice were inoculated subcutaneously with 5×10^4 TC-1 tumor cells. Two weeks later when the tumor volume reached 100 mm^3 (day 0), tumor-bearing mice were treated with cisplatin (5mg/kg body weight) intravenously for three times at 5 days interval. On the same day of the last chemotherapy, mice were vaccinated subcutaneously with micelle vaccine or normal saline (NS) for three times at 7days interval. Tumor growth and tumor free rate was monitored twice per week.

Vaccination combined with surgical operation

C57BL/6 mice were injected subcutaneously on the right flank with 5×10^4 TC-1 tumor cells. One month later when the volume of tumor reached 1000mm^3 (day 0), mice were anesthetized and full resection was executed by excising the tumor using standard blunt dissection. Sterile silk sutures were used to close wounds. Accompanied by wound closure, mice were vaccinated subcutaneously with micelle vaccine or NS for three times at 7 days interval. On the day 42 and 132 after tumor

resection, mice were re-challenged by subcutaneous inoculation of 2×10^5 and 5×10^5 TC-1 cells, respectively. Tumor free rate was monitored twice per week.

1 Zinchuk, V. & Grossenbacher-Zinchuk, O. Recent advances in quantitative colocalization analysis: focus on neuroscience. *Prog Histochem Cytochem* **44**, 125-172, doi:10.1016/j.proghi.2009.03.001 (2009).

2 Labeur, M. S. *et al.* Generation of tumor immunity by bone marrow-derived dendritic cells correlates with dendritic cell maturation stage. *Journal of immunology* **162**, 168-175 (1999).
